# The Sensory Consequences of Speaking: Parametric Neural Cancellation during Speech in Auditory Cortex

**DOI:** 10.1371/journal.pone.0018307

**Published:** 2011-05-19

**Authors:** Ingrid K. Christoffels, Vincent van de Ven, Lourens J. Waldorp, Elia Formisano, Niels O. Schiller

**Affiliations:** 1 Leiden Institute of Brain and Cognition (LIBC) and Leiden Institute of Psychological Research, Leiden University, Leiden, The Netherlands; 2 Department of Cognitive Neuroscience, Faculty of Psychology and Neuroscience, Maastricht University, Maastricht, The Netherlands; 3 Psychological Methods, Department of Psychology, University of Amsterdam, Amsterdam, The Netherlands; 4 Netherlands Institute for Advanced Study (NIAS) in the Humanities and Social Sciences, Wassenaar, The Netherlands; The University of Western Ontario, Canada

## Abstract

When we speak, we provide ourselves with auditory speech input. Efficient monitoring of speech is often hypothesized to depend on matching the predicted sensory consequences from internal motor commands (forward model) with actual sensory feedback. In this paper we tested the forward model hypothesis using functional Magnetic Resonance Imaging. We administered an overt picture naming task in which we parametrically reduced the quality of verbal feedback by noise masking. Presentation of the same auditory input in the absence of overt speech served as listening control condition. Our results suggest that a match between predicted and actual sensory feedback results in inhibition of cancellation of auditory activity because speaking with normal unmasked feedback reduced activity in the auditory cortex compared to listening control conditions. Moreover, during self-generated speech, activation in auditory cortex increased as the feedback quality of the self-generated speech decreased. We conclude that during speaking early auditory cortex is involved in matching external signals with an internally generated model or prediction of sensory consequences, the locus of which may reside in auditory or higher order brain areas. Matching at early auditory cortex may provide a very sensitive monitoring mechanism that highlights speech production errors at very early levels of processing and may efficiently determine the self-agency of speech input.

## Introduction

Speaking includes the perception and monitoring of one's own speech for errors, which requires distinguishing self-generated acoustic signals from external signals [1], [2]. Classic as well as contemporary neuroscience research suggests that monitoring of self-generated speech relies on the interaction between motor and sensory processing systems, in which forward models of vocal commands predict the sensory consequences of speaking [3], [4]. Forward models are considered to play a substantial role in general motor control. The forward model predicts the next state of a process given the current state and the motor command [5]. According to Wolpert et al [5], theoretically, the forward model has a number of uses, for example that it allows for the outcome of any action or intention to act to be estimated and used before the actual sensory feedback becomes available, and it may provide information on the desired versus actual outcome that is crucial to motor learning. In the present paper the most relevant aspect of the forward model is that it can be used to anticipate and cancel the sensory effects of our own actions [5]. This provides for example a compelling explanation for why we cannot tickle ourselves [6].

The forward model framework applied to speech production suggests that a copy of speech motor commands (‘efference copy’) allows for the prediction of sensory consequences of motor commands. This prediction is then compared to the actual sensory feedback, and mismatches between actual and predicted auditory signals are neurally encoded. Such a mismatch may occur in cases of speech production errors, but also when speech feedback quality is impaired. For example, this could be the case in a loud or noisy environment. For speech the comparison between predicted an actual verbal feedback makes it possible to distinguish our self-generated speech input from external speech, a function that is crucial for example in conversations.

Brain imaging studies provide support for predictions of a forward model during speech production. Functional magnetic resonance imaging (fMRI) and positron emission tomography (PET) studies in human participants presented modulated verbal feedback by manipulating the pitch of speech [7], [8], [9], delaying feedback [10], or superimposing feedback noise masks [4], [11], [12]. These studies showed that impaired or modulated feedback compared to normal self-generated feedback resulted in increased activity in auditory regions of the superior temporal gyrus (STG). Importantly, these findings have been interpreted as evidence for reduced activity during unimpaired self-generated feedback [11] but also, in contrast, as increased activity during altered feedback compared to self-generated feedback [8], [13]. Thus, while these results appear to support the forward model, the two different interpretations indicate different mechanisms underlying speech monitoring in sensory cortex. Specifically, the first interpretation suggests a mechanism in which neural activity is locally cancelled or inhibited as a consequence of a match between predicted and actual speech feedback. A match may decrease neural activity in auditory areas activated by verbal feedback. In case of a mismatch no such reduction of auditory activity takes place when speaking. The second interpretation entails that altered speech feedback increases activity compared to normal speech monitoring, for example, due to auditory error signaling cells in auditory cortex during manipulated feedback [8].

Findings from neurophysiological recordings in monkeys [14], [15] as well as from electrophysiological recordings on the scalp in humans [16], [17], [18] have shown decreased activity in response to self-generated vocalizations, suggesting that feedback results in inhibition of activity in sensory areas. The relationship between the hemodynamic response and neural activity is not completely clear [19]. Indeed, in most fMRI studies that report increased BOLD response during impaired feedback compared to normal feedback, this pattern has been interpreted as increased activity during modulated feedback, due to increased monitoring effort or error coding [8], [12].

A confound in this discussion is the definition of the baseline condition in fMRI. Within the forward model framework the sensory response to externally generated acoustic signals should be regarded as the baseline, during which the motor system does not provide any sensory prediction. However, most studies compare normal and altered feedback during self-generated speech. In both conditions the forward model is generated which contains a sensory prediction. This provides an ambiguous comparison in terms of increased or decreased activity in speech monitoring. Therefore, strictly speaking, these studies do not test the forward model. In a previous fMRI study, we included a listening baseline condition to test the forward model [11]; see also [12] for a recent replication of those results. In these studies, participants listened either on-line to their own overt speech or to a previously made recording of their own voice played back to them. At the same time, in both conditions the speech was masked by noise to manipulate the quality of feedback. Our results showed that brain activity in bilateral superior temporal gyrus decreased during monitoring of self-generated speech, compared to unmasked listening. Furthermore, the noise mask superimposed on self-generated speech increased auditory cortex activity to the level of masked and unmasked pre-recorded speech. We interpreted these findings as attenuated activity during normal feedback. The pattern is consistent with the neural cancellation of sensory activity predicted by the forward model framework. However, our previous study, as well as other fMRI studies of speech monitoring [e.g.], [ 7], [8], [12,13] used an ON-OFF design. Such designs do not warrant conclusions with respect to the hypothesis of neural cancellation. A stronger test of the forward model is to use a parametric design to systematically vary the feedback quality, which allows the investigation of the specificity of the predictions of the forward model. Specifically, a strong prediction of the neural cancellation hypothesis is that sensory cortical activity should increase gradually with decreased feedback quality during self-generated speech. In other words, signal reduction should be a function of the degree of mismatch between predicted and perceived speech feedback.

In the current fMRI study, we investigate whether a match of predicted and actual feedback indeed results in net inhibition and as a consequence in reduced activity during speaking. Furthermore, we investigate the specificity of the predictions of the motor commands. We used a parametric design of feedback masking to address these two issues. During picture naming, participants either listened to their self-generated speech or they watched pictures and listened to their own pre-recorded speech on-line while a noise mask was superimposed at different intensity levels. Intensity levels ranged from zero to a maximum level at which participants could no longer hear their own speech. We predicted that unmasked verbal feedback resulted in decreased auditory cortex activity compared to listening to prerecorded speech. We further predicted that activity of auditory cortex increased parametrically with decreasing quality of verbal feedback during speaking. Finally, we predicted that the parametric noise mask did not alter auditory cortex activity during listening to pre-recorded speech.

## Methods

### Participants

Eleven healthy volunteers (3 male, 8 female, mean age 22.3 years; range 19–26) without any history of neurological or psychiatric disease participated in this study. They were right-handed according to the Edinburgh Handedness Inventory [19] Participants were undergraduate or graduate students at Maastricht University, native speakers of Dutch and had no history of hearing or language related problems. All participants gave their written informed consent. The study was approved by the Ethical Committee of the University Medical Center of Maastricht, the Netherlands.

### Stimulus material and task design

**Figure 1:**
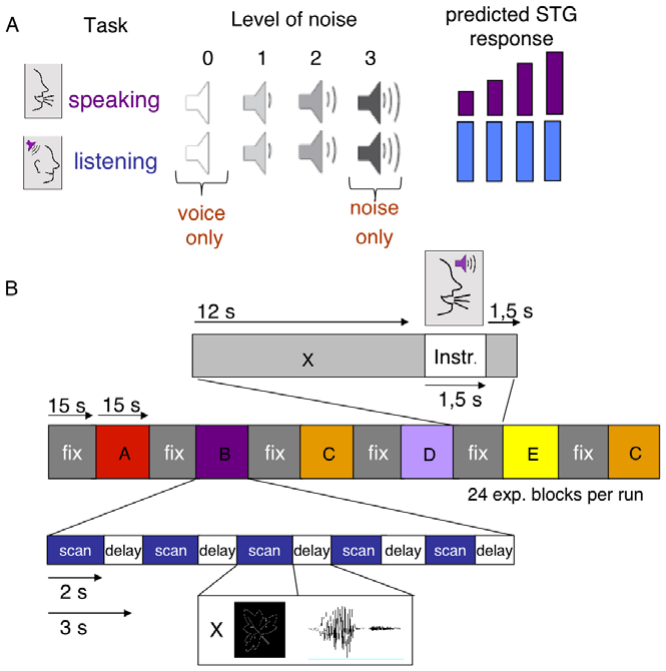
Design and expectations. **A**. The intensity of the noise was parametrically varied from no noise to loud masking noise. Loudspeaker icons (x-axis) indicate the noise level, from zero (white loudspeaker) to maximal noise masking (black loudspeaker). The BOLD response was predicted to be attenuated as a consequence of feedback quality during speaking not listening (auditory control) in superior temporal gyrus (STG). **B**. Stimulation protocol.

We used a 2 (task)×4 (noise level) factorial blocked design (see **Figure 1**). The two tasks consisted of overt speaking (picture naming, PN) and passive listening (LIS) to pre-recorded speech while watching pictures. In the speaking conditions, participants were required to overtly name visually presented pictures. In the listening conditions, participants were asked to passively view pictures and listen to their own pre-recorded picture naming responses. During PN and LIS conditions, externally presented acoustic noise was superimposed on the self-generated speech feedback and pre-recorded speech respectively. The noise level was parametrically varied across four intensity levels, with the first level set to zero intensity and the fourth level to maximum intensity. Noise levels were crossed with the tasks to obtain eight experimental conditions: voice only (PN0, LIS0), voice combined with relatively soft noise (PN1, LIS1), voice combined with louder noise (PN2, LIS2), and loud masking noise (PN3, LIS3).

The superimposed noise sound consisted of 1,500 ms of digital mono recording of pink noise (1993, Sound Check Productions, A. Parson and S. Court). In the LIS conditions, the same pink noise recording was presented with the same timing as the PN conditions. Due to individual differences in voice and sensitivity to sound, the noise volume was determined separately for each participant before the actual scanning started. We adjusted the noise level in each individual subject based on an interactive procedure performed in the scanner before the actual experiment started. After being placed in the scanner, participants were asked to name pictures (that were not used in the actual experiment) while the noise was played. The volume of the noise was gradually increased until participants reported they could no longer hear themselves. Noise intensity was set at a level such that the subject consistently reported not to hear his/her own voice. The final noise volume was taken as maximum noise level intensity (level3). Intermediate intensity levels were obtained by decreasing the maximum level with 10 (level2) and 15 dB SPL (level1). This procedure resulted in an approximate level of noise intensity of 102 dB SPL on average (measured outside the scanner room). Given the subjects' own reports, the levels of noise employed, the approximate volume of subjects' voice (40–50 dB SPL), we may conclude that the subjects could not hear themselves in the loudest noise condition, irrespective of bone conduction. We next gave example trials of both speaking and listening conditions without added noise and asked the participants, again using an interactive procedure to subjectively equate levels of their own spoken input and the presented speech. Apart from the different noise levels used, the experimental protocol was similar to the one used in Christoffels et al. [11]. In the picture-naming conditions, participants were required to name pictures as quickly and accurately as possible. Participants were instructed not to be concerned with audibility to minimize speech-related movement; they were told not to over-articulate or speak loudly. Furthermore, they were made aware of the automatic tendency to increase the loudness of their voice in the presence of noise (Lombard effect [20]) and were instructed to speak at the same volume throughout the experiment. Audio recordings made during the experiment indicated that the responses were of the same volume in each of the speaking and the listening conditions, which suggests that results cannot be explained by the Lombard effect.

Each experimental block consisted of five trials of one of the eight conditions and lasted 15 s. Experimental blocks were interspersed with a fixation block that lasted 15 s, in which a symbolic instruction was visually presented for 1,500 ms to cue the task in the upcoming experimental block at 12 s after fixation block onset. Conditions were presented in four functional runs. Each run consisted of 24 experimental task blocks that alternated with fixation blocks. There were three repetitions of each condition per run, adding to a total of twelve repetitions of each condition for each participant. The block order varied pseudo-randomly between runs and run order was counterbalanced across participants. In all conditions, trials started with the presentation of a fixation cross for 1,550 ms, followed by the presentation of a picture for 1,000 ms. We chose stimulus presentation timing in such a way that the picture onset was 450 ms before the onset of the quiet interval between volume acquisitions (see Imaging Parameters) because participants needed at least this time to generate the response. In the PN conditions, participants responded in the next 1,000 ms of silence before the next functional volume was acquired. Presentation of the 1,500 ms noise was synchronized to picture presentation, which therefore covered the quiet interval between volume acquisitions. In the LIS conditions, presentation of the auditory picture word started 700 ms after picture presentation (i.e., 250 ms into the quiet interval). See **Figure 1B** for a graphical presentation of the stimulation protocol.

During each functional run, a digital audio recording was made of the participants' verbal responses. The audio-recordings indicated that the responses were of the same volume across the PN conditions.

The pictures comprised twenty simple white-on-black line drawings, which were presented equally often in each condition across runs. Pictures from the Max Planck Institute for Psycholinguistics database were selected for high name agreement (mean = 99.1%; the percentage in which a given picture solicited the same name across participants, pre-tested in a pilot study with different native Dutch participants). Picture names corresponded to mono- and bisyllabic words of relatively high word frequency (on average 223 occurrences per one million words, CELEX database and were 3.75 phonemes long on average). For the LIS conditions, picture-naming responses were recorded for each participant in a separate session in a soundproof booth prior to the scanning session. This resulted in 20 unique auditory stimuli, which were presented in all listening conditions: i.e., one recording for each picture (44.1 kH, 16 bits, mono), for each participant.

Prior to the fMRI experimental runs, a localizer run was administered with two PN conditions (PN0 and PN3), using ten novel pictures not used in the main experiment. In other respects the localizer run was identical to the experimental runs.

### MR Imaging parameters

Imaging was performed on a 3 Tesla head scanner (Magnetom Allegra, Siemens Medical Systems) located at the Maastricht Brain Imaging Center (M-BIC). Functional volumes were acquired using a T2*-weighted echoplanar sequence with blood oxygen level-dependent (BOLD) contrast (TR = 3 s, TE/TRslice = 30/60 ms, slice thickness = 3 mm, interslice distance = 0.5 mm, matrix = 64×64, number of slices = 34, voxel size = 3.5×3.5×3 mm^3^). We used a sparse sampling procedure for functional data [11], [21], in which functional data of each whole-brain scan were acquired in a 2 s time window, followed by an interscan gap of 1 s. High-resolution structural scans (voxel size 1×1×1 mm^3^) were acquired using a T1-weighted 3D “modified driven equilibrium Fourier transform” (MDEFT) sequence (192 sagittal slices, TR = 7.92 ms, TE = 2.4 ms).

Participants were placed comfortably in the scanner with their heads fixated using the headset and foam pads. Mounted on the head coil was a mirror through which participants could see the stimuli projected on a screen placed outside the scanner. Auditory stimuli were presented through an MR-compatible Intercom Commander XG MRI Audio System (Resonance Technologies Inc.) using a 2-way stereo headset. The headset served as ear defender but did not prevent participants from hearing their own voice in unmasked feedback conditions. Prior to scanning the volume of the noise was set individually via the audio system. An audio recording of the participant's responses was made for each run with a microphone attached to the headset. The presentation of each trial was synchronized with MR data acquisition by using an MR pulse to trigger each trial. Recordings of the participants' responses were checked for correctness in the speaking conditions and for lack of any responses in listening conditions, which revealed no naming errors and a very small number of missing responses (0.49%). During debriefing participants consistently reported that they did not hear their own voice during the loudest noise conditions, confirming that noise masking was successful.

### Data Analyses

#### Pre-processing

Anatomical and functional images were analyzed using BrainVoyager QX (Brain Innovation, Maastricht, The Netherlands). The first four functional volumes of each run were discarded to take the T1 saturation effect into account. The pre-processing steps of the functional images included slice scan time correction (using sinc interpolation), three-dimensional (3D) motion correction (least squares using the Levenberg-Marquardt algorithm) to detect and correct for small head movements by spatially aligning the volumes of all functional (experimental and localizer) runs of each participant to the first volume of the first experimental run, linear trend removal and temporal high-pass filtering to remove drifts of three or fewer cycles per time course (i.e., ≈0.004 Hz) [22]. The estimated translation (x, y and z) and rotation parameters (roll, pitch and yaw) that resulted from the motion correction were inspected and never exceeded 3 mm or degrees within each run. No spatial smoothing was applied to the data. Pre-processed functional time-series were co-registered to the within-session anatomical 3D dataset using position parameters from the scanner and manual adjustment. Anatomical and functional data were then transformed into Talairach space [23] and re-sampled to an iso-voxel resolution of respectively 1×1×1 and 3×3×3 mm^3^. Pre-processed and Talairach-normalized functional volume time-series were used for the statistical analysis.

### Auditory cortex activity to parametric feedback

For analysis of brain activity related to speech feedback we used a canonical hemodynamic response function (HRF; combination of two gamma functions) in a general linear model (GLM) [24]. We used the localizer run to select areas in the temporal cortex in order to include only relevant regions in the main analysis. We performed a two-level GLM (similar to a random-effects [RFX] analysis [24] to test the contrast between speaking without and with noise (i.e., PN0<PN3). In the first level, regression (beta) coefficients for each of the two conditions were estimated for each participant. In the second level, for each participant the contrast [PN0–PN3] was calculated, and the distribution of contrast values across participants was tested to be different from zero using a one-sample t-test (df = 10). The t-values were superimposed on the anatomical images and visualized using an uncorrected statistical threshold of 0.05. Voxels that survived the threshold were tagged to create a spatial mask to restrict the voxel-based analysis of the experimental runs. This way, we applied our spatial hypothesis that specific areas in bilateral STG showed parametric effects to noise masking to the experimental runs, and reduced the size of the multiple comparison problem [25], [26].

Second, we analyzed the time series of the experimental runs of the localizer-tagged voxels using a 2 (PN and LIS)×4 (Noise level 0, 1, 2 and 3) two-level RFX GLM. In the first level of the analysis, we calculated the beta coefficients for the eight conditions for each participant. In the second level, we tested the parametric contrast of [−3, −1, 1, 3] on the four levels of the PN condition across participants to specifically test for the predicted parametric pattern of decreasing BOLD signal amplitude with increasing quality of verbal feedback. Results were superimposed on the anatomical images and visualized using a Bonferroni-corrected threshold of p = 0.05 to correct for multiple comparisons. We used a repeated measures analysis of variance (ANOVA) of the beta coefficients of the voxels that were significant for the contrast to test for the expected interaction between speaking and listening.

## Results

### Localization and parametric effect

**Figure 2:**
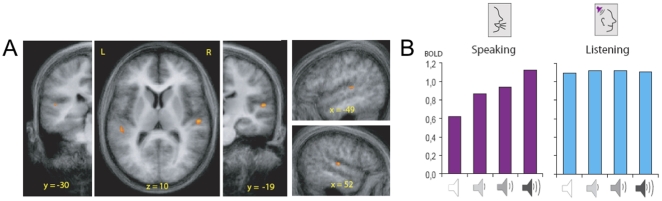
Localization and contrast results. **A**. The random effect results of the auditory cortex (localized using independent functional runs for each participant), thresholded at p<.05 (Bonferroni corrected) are superimposed on a three-dimensional anatomical image (average of all participants). Clusters in left and right STG were significantly activated for the contrast tested for speaking conditions. **B**. The peak of the fMRI response (% signal change) plotted for speaking and listening conditions for the voxel with the maximum statistic for the parametric contrast (Coordinates x = 51, y = −19, z = 10). Loudspeaker icons (x-axis) indicate the noise level, from zero (white loudspeaker) to maximal noise masking (black loudspeaker). Repeated measurement ANOVA confirmed a significant Task×Noise interaction effect of the peak responses (repeated measures ANOVA; F(3,30) = 11.4, P<0.001).

The RFX analysis of the localizer run resulted in a mask of auditory cortex that included bilateral Heschl's gyrus and sulcus, as well as anterior parts of the superior temporal plane. We used the mask from the localizer run to restrict the number of voxels included in the analyses of the main experiment. Here, the statistical parametric contrast of main interest of noise in picture naming in independently localized auditory cortex yielded three significant clusters within and around left Heschl's sulcus and right planum temporale (PT) (**Figure 2A**). The largest cluster was obtained in the right STG (center of gravity: x = 52; y = −20; z = 10; size = 110 mm^3^). Further, we found a second area in the right STG, located posterior to the first (x = 43; y = −28; z = 13, size = 45 mm^3^) and in left STG at or close to Heschl's sulcus (x = −47, y = −30; z = 9, size = 37 mm^3^). The average amplitudes of the maximum-statistic voxel time-locked to the PN and LIS blocks across the four noise levels revealed the predicted parametric pattern (**Figure 2B**), i.e., during overt speech, signal amplitude increased with decreasing feedback quality. At the maximum noise level, the response to feedback in the speaking condition was similar to the response to passive listening. This implies that auditory cortex activity decreased during normal speech feedback. Crucially, there was no evidence for a parametric response in amplitude for the auditory input during passive listening, showing that our results in the speech condition were not due to qualitative differences in noise masking. Indeed, in the relevant voxels we also tested the parametric contrast for the LIS conditions. Here, results were not significant for any voxel in the clusters, even at a more lenient statistical threshold (p<.001, uncorrected). We summarized these effects using a repeated measures analysis of variance of the beta coefficients of the voxels that showed the parametric effect (**Figure 2A**). We found a significant Task×Noise interaction effect for the time series of the parametric voxels (F(3,30) = 12.3, p<0.001), in addition to significant main effects for Task (F(1,10) = 12.4, p = 0.005) and Noise (F(3,30) = 5.5, p = 0.004). In other words, for the listening conditions, the regions did not respond parametrically to the increase in masking noise.

We also tested for the parametric effect in a whole-brain RFX analysis that was not restricted by the auditory cortex mask. We did not find any significant voxels for the parametric effect in this analysis.

## Discussion

In the current study we parametrically manipulated feedback quality during self-generated and pre-recorded speech. We investigated the specificity of the predictions of the forward model of speech monitoring and assessed whether the consequences of a graded mismatch results in graded net inhibition or activation. We found that activity in early auditory cortical regions varied proportional to the parametric level of the noise mask during overt speech. The forward model prediction appears to be very specific. This suggests that modulation of cortical activity by the forward model may be a very sensitive mechanism, which may highlight even small speech production errors and may easily distinguish between self-generated speech and external speech.

Modulation of activity in STG due to a feedback manipulation has been reported in previous studies [e.g.], [ 4], [8], [11], [13,18]. However, in contrast to previous studies we showed that this modulation could be parametrically manipulated, indicating that the degree of match between predicted and actual feedback determines the amount of attenuation. Secondly, we demonstrated that the parametric effect was absent during the passive listening conditions. Finally, signal amplitude of auditory cortex activity during passive listening was similar to the amplitude of auditory cortex during overt speech being masked by the highest noise level. This is important because it provides the baseline to which unimpaired feedback can be compared: Our results clearly indicate that the modulation of auditory cortex found in previous studies should be interpreted as reduction or inhibition of neural activity due to the match between expected and perceived feedback, rather than increased activity due to altered feedback [7], [8], [13]. Further, our results are in line with neurophysiological recordings in animals [14], [15] and electrophysiological recordings in humans [16], [17], [18] that showed attenuated activity in auditory cortex during self-generated vocalizations or overt speech. These findings suggest that neural cancellation takes place in auditory cortex as a function of feedback quality.

We observed that only relatively small regions in the bilateral early auditory cortex responded to feedback parametrically. These regions are similar to the regions obtained by other studies using listening control conditions [11], [12]. Interestingly, these regions have been indicated as relevant sites for interfacing auditory and motor representations and have been associated with interfacing of multimodal representations, including auditory and somatosensory [27], [28], [29]. Overall, our and other findings suggest that early auditory cortex may be associated with top down processing of auditory signals, rather than being restricted to bottom up acoustic processing.

Our study revealed no parametric activity in brain areas outside of the auditory cortex. A number of previous monitoring studies did report activity in other brain areas when comparing normal versus altered feedback, such as the anterior cingulate cortex and supplementary motor area [11], [26]. We argue that these non-auditory areas may not be involved in assessing the degree of mismatch. Motor areas provide the sensory predictions of the forward model and information about the (mis)match may be forwarded for adaptive processing on errors in further speech production. Thus, while motor areas may provide a prediction of the sensory consequences of overt speech, early auditory processing areas could be involved in the comparison of perceived speech and predicted feedback.

Our findings may have important implications for understanding speech-related deficits. For example, auditory activation by one's own voice in people who stutter has been found to be weaker compared to healthy control subjects [30]. This may severely disturb the proper working of self-monitoring in speech, since our finding suggests that in localized regions in the auditory cortex important matching functions are performed. In addition, our finding of the role of auditory cortex in speech monitoring may also be of importance to understanding auditory verbal hallucinations in schizophrenia and other psychotic disorders, which has been attributed to impaired speech monitoring [31]. This may be manifested by increased activity in early auditory regions during the perception of spontaneously occurring auditory verbal hallucinations [25], [32], as opposed to a lack of activity in these regions during voluntarily controlled auditory imagery of speech in healthy controls [11], [33], [34] as well as hallucinating patients [35]. In this sense, our finding suggests that impaired monitoring of speech may lead to increased auditory cortex activity during self-generated auditory images, which could in pathologies lead to hallucinatory experiences.

An important implication of our study is that neurophysiological activity in auditory cortex is inhibited during unaltered speech feedback. Although we could not measure neurophysiological responses in our study, our results are consistent with electrophysiological recordings in humans and animals that showed inhibition of auditory neurons when listening to self-generated vocal sounds or speech [14], [15], [36], [37]. Further, previous studies have provided empirical evidence that the fMRI signal may be highly correlated to field responses of groups of neurons [19], [38]. Future studies that can combine neurophysiological with non-invasive brain imaging techniques may be able to elucidate the exact neurophysiological mechanism of neural cancellation in auditory cortex during overt speech.

In conclusion, using fMRI we were able to demonstrate a parametric attenuation of auditory cortex response in humans during overt speaking, visible as net reduction in BOLD response in auditory cortex. From our findings, it is clear that a forward model makes a very specific prediction of the expected self-generated auditory input resulting in neural cancellation in bilateral auditory cortex.
